# Untargeted metabolomics reveals the mechanism of amantadine toxicity on *Laminaria japonica*

**DOI:** 10.3389/fphys.2024.1448259

**Published:** 2024-07-24

**Authors:** Xiaohan Wang, Yao Lu, Jinxia He, Xiaojie Li, Yingjiang Xu, Lihua Ren, Huanjun Li

**Affiliations:** ^1^ Yantai Key Laboratory of Quality and Safety Control and Deep Processing of Marine Food, Shandong Key Laboratory of Marine Ecological Restoration, Shandong Marine Resource and Environment Research Institute, Yantai, China; ^2^ Shandong Oriental Ocean Technology Co. Ltd., Yantai, China

**Keywords:** amantadine, Laminaria japonica, metabolites, toxicological effect, phospholipid metabolism, amino acids

## Abstract

The antiviral agent amantadine is frequently detected in seawater and marine organisms. Because of increasing concentrations, amantadine has become a contaminant of emerging concern. This compound has toxic effects on the brown algae *Laminaria japonica*. The effects of amantadine on the biological processes of *L. japonica* and the corresponding toxic mechanisms remain unclear. In this study, amantadine toxicity on *L. japonica* was investigated using histopathological and physiological characteristics combined with metabolomics analysis. Changes in metabolites were determined by untargeted metabolomics after exposure to 10^7^ ng/L amantadine for 72 h. The catalase activity in the exposure group slightly increased, whereas the superoxide dismutase activity greatly decreased. An increase in the malondialdehyde concentration was observed after amantadine exposure, which suggested that lipid peroxidation and cell damage occurred. Metabolomics analysis showed that there were 406 differentially expressed metabolites after amantadine exposure. These were mainly phospholipids, amino acids, purines, and their derivatives. Inhibition of the glycerophospholipid metabolism affected the lipid bilayer and cell structure, which was aligned with changes in histological observation. Changes in amino acids led to perturbation of protein synthesis and induced oxidative stress through interference with glutathione metabolism and tyrosine metabolism. Amantadine also interfered with energy metabolism in *L. japonica* by disturbing the tricarboxylic acid cycle and purine metabolism. The results of this study provide new insights into the mechanism of amantadine toxicity on *L. japonica*.

## 1 Introduction

Brown algae, including *Laminaria japonica*, *Laminaria digitata*, *Undaria pinnatifida*, *Macrocystis pyrifera*, *Sargassum natans*, and others, are a large group of marine seaweeds (∼1800 species) with high biomass (Wei et al., 2013; Shen et al., 2021; Sun et al., 2023). *L. japonica*, also known as kelp, is one of the most widely consumed seafoods in China and many other nations because of its content of substances with high biological activity (Luan et al., 2021). In 2021, China produced 1.74 million tons of kelp, with a year-on-year growth rate of 5.50% (China Agriculture Department, 2022). However, the marginal production of kelp is gradually declining in China according to kelp industry development report (China academic journal electronic publishing house, 2021), which could be caused by global warming and water pollution. Recently, the widespread use of pesticides, heavy metals, and pharmaceuticals in anthropogenic activities has caused their continuous emission into aquatic ecosystems (Zhang et al., 2021; Hejna et al., 2022). These aquatic pollutants pose a serious threat to marine ecosystems and have become a worldwide problem (Nikolaou et al., 2007; Prichard and Granek, 2016). *L*. *japonica* and other macroalgae are often exposed to environmental conditions that can lead to accumulation of various pollutants in their tissues, including toxic metals (arsenic, cadmium, lead, and mercury), pharmaceuticals (salicylic acid, paracetamol, carbamazepine, and atenolol), and pesticides (terbuthylazine, metazachlor, and picoxystrobin) (Xiao et al., 2012; Claessens et al., 2013; Bighiu et al., 2020; Garcia-Vaquero et al., 2021). The negative effects of these pollutants have been studied. For instance, because cadmium dysregulates the enzymes involved in carbohydrate and energy metabolism, it inhibits metabolic activity in *Sargassum fusiforme* (Zhang et al., 2015). Research has shown that heavy metal pollutants can cause damage to algae, and this mainly occurs through oxidative stress (Wang et al., 2023).

Amantadine, a stable and water-soluble quasi-spherical cyclic primary amine, has been used for Parkinson’s disease treatment and can also be used in aquaculture to control influenza A virus (Ma and Zafonte, 2020; Raharja et al., 2023). Nevertheless, the illegal overuse of amantadine in aquaculture has caused pollution in aquatic environment, especially in coastal water and surface seawater (Peng et al., 2019; Zhang et al., 2021). In the surface seawater of Jiaozhou Bay (China), amantadine is one of the most abundant pharmaceutically active compounds (12.6–73.6 ng/L) with a detection frequency of 100% at all sampling sites (Peng et al., 2019). Amantadine has also been detected in seawater from mariculture areas in Northeast China at concentrations ranging from 15 to 140 ng/L (Zhang et al., 2021). Furthermore, the amantadine remained in marine environment is absorbed and bioaccumulated by plants and aquatic animals such as macroalgae, barnacles, and fish (Zeng et al., 2022). In *L. japonica* from Daqin Island (China), amantadine has been detected at 8.26–17.6 μg/kg, which indicates a bioenrichment effect (Xu et al., 2019). The adverse effect of amantadine on aquatic organisms have been reported by numerous studies and raised increasing concern in recent decades (Xiang et al., 2021). Scalzo et al. (2011) confirmed that zebrafish embryos exposed to high concentrations of amantadine exhibited signs of malformations, including edema and scoliosis. Zhao et al. (2023) reported that amantadine triggered oxidative stress, inflammation, and apoptosis in *Apostichopus japonicus*.

The side effects of amantadine on human health include cellular apoptosis, poisoning and tissue damage (Ma and Zafonte, 2020; Barbara and Pace, 2023), but studies on the toxic mechanisms of amantadine on *L. japonica* have been limited. Because of the adverse reactions and toxicity of amantadine, it is important to understand the biological effects and toxic-response mechanisms of amantadine on *L. japonica*. Untargeted metabolomics has been widely used to elucidate the toxic mechanisms of pollutants because it is rapid, accurate, and efficient for the analysis of changes in metabolites (Song et al., 2018; Xu et al., 2020). Felline et al. (2019) used a metabolomics approach and found that glyphosate resulted in variation in the levels of aromatic amino acids and other metabolites and significantly reduced the quantum yield of photosynthesis in the brown algae *Fucus virsoides*. Shen et al. (2021) used metabolomics to evaluate differences in the concentration and variety of polyphenolics in four types of brown macroalgae, including *L. japonica*. These studies suggest that metabolomics will be effective for exploring the toxic mechanisms of amantadine on *L. japonica*.

In this study, the effects of amantadine on *L. japonica* were investigated at an exposure concentration of 10^7^ ng/L. The antioxidant capacities of *L. japonica* were estimated by detecting the superoxide dismutase (SOD) activities and catalase (CAT) activities, and malondialdehyde (MDA) concentrations were used to evaluate the oxidative damage induced by amantadine stress. Untargeted metabolomics was used to study metabolic changes in *L. japonica* exposed to high levels of amantadine, and functional annotation and enrichment analysis of the detected metabolites were performed. This research provides a basis for identifying the toxic-response mechanisms of *L. japonica* to amantadine exposure and developing strategies for preventing amantadine pollution in seawater.

## 2 Materials and methods

### 2.1 Experimental materials

In previous acute toxicity studies, the aquatic organisms were exposed to different concentrations of amantadine (ranging from 100 μg/L to 400 mg/L) (Scalzo et al., 2011; Zhao et al., 2023). In this study, juvenile sporophytes of *L. japonica* were exposed to 10^7^ ng/L of amantadine for 72 h. This exposure condition was conducted following the methods previously reported with minor modifications. Under this exposure condition, the samples in amantadine treatment group (AT) exhibited significant growth inhibition compared with the control group (CG).

Juvenile sporophytes of *L. japonica* were obtained from Oriental Ocean Technology Co., Ltd (Yantai, China). Seedling ropes (length: 50 ± 2 cm) were acclimated in seawater for 3 days. Afterwards, seedling ropes (n = 16) with similar densities were randomly selected for the exposure experiment. Amantadine (98% purity) was purchased from Shanghai Maclean Biochemical Technology Co., Ltd (Shanghai, China), and dissolved directly in seawater to prepare a 100 mg/L stock solution. The sporophyte samples in CG and AT were maintained in seawater containing 0 or 10^7^ ng/L amantadine, respectively. Each treatment group comprised eight replicates. The seedling ropes (fixed with suction cups) were kept in a laboratory aquaculture tank (40 L) filled with 30 L of continuously oxygenated seawater. The water temperature was 10°C ± 1°C and light intensity was 5000 lux with a 12:12 h light-dark photoperiod. Nutrient salt containing nitrogen (10 mg/L) and phosphorus (1 mg/L) was added to the seawater. The seawater was exchanged on the third day over the 5 days of exposure, and the amantadine stock solution was supplemented to maintain the experimental concentration.

### 2.2 Sample preparation

After 72 h of exposure, 2 g of juvenile sporophytes of *L. japonica* from each parallel sample were selected and equally divided into two cryotubes for metabolomics analysis. On days 0, 3 and 5, three samples from both the CG and AT group were randomly selected for enzymatic activity analyses. After collection, the samples were snap-frozen in liquid nitrogen and stored at −80°C.

### 2.3 Histological and biochemical response

On days 1 and 3, three parallel samples of leaf tissue (1 × 1 cm) were selected randomly from both the CG and AT group. These samples were fixed in a 10-fold volume of Bouin’s solution for 24 h and then preserved in alcohol (70%, v/v). The fixed samples were subsequently dehydrated in alcohol, made transparent in xylene, submerged in wax, and embedded in paraffin using an embedding machine. After trimming with a microtome, the slices were baked at 60°C, stained with a hematoxylin and eosin (H&E) automatic staining machine, and sealed with neutral gum. The samples were observed under a light microscope and photographed for documentation. To determine the enzyme activities, the samples were cut into pieces and mixed with phosphate-buffered saline (pH 7.4) at a 1:4 mass-to-volume ratio and homogenized in an ice bath using a portable high-speed disperser. After centrifugation, the supernatant was collected to determine the CAT activities, SOD activities, and MDA concentrations using assay kits purchased from the Nanjing Jiancheng Institute of Biological Engineering (Nanjing, China). The absorbance was detected by a microplate reader. The enzyme activities are shown as fresh weight (FW).

### 2.4 Metabolite extraction

To extract pure metabolites, 50 mg of samples were accurately weighed into a 2-mL centrifuge tube, and then homogenized in 400 μL of extraction solution (methanol/water, 4:1 v/v) using a high-throughput tissue crusher (Wonbio-96c, Shanghai Wonbio Biotechnology Co., Ltd., Shanghai, China) at −10°C for 6 min l-2-Chlorophenylalanine with a concentration of 0.02 mg/mL was used as the internal standard. Subsequently, the samples were treated by ultrasonic extraction for 30 min (40 kHz, 5°C) and then kept at −20°C for 30 min. After centrifugation at 13,000 ×*g* for 15 min at 4°C, the supernatant was transferred to an autosampler vial with an insert for ultra-performance liquid chromatography-time-of-flight-mass spectrometry (UPLC-TOF/MS) analysis.

### 2.5 Untargeted metabolomics analysis

Quality control (QC) samples were prepared by mixing 20 μL of the supernatant from each test sample and were then processed and analyzed using the identical method as the other samples. Throughout the instrumental analysis process, the QC samples were analyzed every eight samples to evaluate the stability of the detection process.

UPLC-TOF/MS was conducted following established methods (Mwamba et al., 2020; Shen et al., 2021). Chromatographic separation of the metabolites was performed using UPLC-TOF/MS (AB SCIEX) with an ACQUITY HSS T3 UPLC column (100 mm × 2.1 mm i.d., 1.8 μm; Waters, Milford, United States). Mobile phase A was ultrapure water containing 5% acetonitrile and 0.1% formic acid, and mobile phase B was a mixture of 47.5% acetonitrile, 47.5% isopropanol, and 5% water (with 0.1% formic acid). The solvent gradient was as follows: 0–0.5 min, 100% solvent A; 0.5–2.5 min, 100%–75% solvent A; 2.5–9 min, 25%–100% solvent B; 9–13 min, 100% solvent B; 13–13.1 min, 0%–100% solvent A; 13.1–16 min, 100% solvent A for equilibration. The column temperature was maintained at 40°C. The injection volume was 10 μL and the flow rate was 0.4 mL/min. All samples were stored at 4°C during analysis.

Mass spectrometry was conducted in both positive and negative ion modes, and the data was collected using a time-of-flight mass spectrometer equipped with an electrospray ionization source. The parameters were as follows: curtain gas, 30 psi; source temperature, 550°C; ion spray voltage floating (+), 5000V; ion spray voltage floating (−), 4000 V; declustering potential, 80 V; collision energy, 40 ± 20 eV; and cycle time, 510 ms. Data detection was performed over a mass range of 50–1,200 m/z.

The raw data were imported into Progenesis QI (Waters Corporation) to obtain a two-dimensional data matrix of the peak intensity, mass-to-nucleus ratio, and retention time. Metabolic features detected in at least 80% of any set of samples were retained. After filtering, minimum metabolite values were imputed for specific samples in which the metabolite levels fell below the lower limit of quantitation, and each metabolic feature was normalized by sum. The internal standard was used for data QC (reproducibility), and the relative standard deviation of the internal standard exceeded 30%. Following the normalization procedures and imputation, the log-transformed data were statistically analyzed to identify significant differences in metabolite levels between comparable groups. The preprocessing results generated a data matrix that consisted of the retention time, the m/z values, and peak intensities. Metabolic features from the recorded MS data were identified using data from the Human Metabolome database (HMDB) (http://www.hmdb.ca/) and Metlin database (https://metlin.scripps.edu/).

### 2.6 Multivariate statistical analysis

A multivariate statistical analysis was performed on the Majorbio Cloud platform (https://cloud.majorbio.com). To obtain an overview of metabolic data and evaluate the differences between samples, an unsupervised principal component analysis method was performed using ropls (version 1.6.2, http://bioconductor.org/packages/release/bioc/html/ropls.html) R packages from Bioconductor on the Majorbio Cloud platform. Partial least squares discriminant analysis (PLS-DA) and orthogonal partial least squares discriminant analysis (OPLS-DA) were conducted to determine the overall metabolic changes between the CG and AT groups. The variable importance in the projection (VIP) was calculated in the PLS-DA and OPLS-DA models using ropls and scipy.status (Python packages, version 1.0.0, https://docs.scipy.org/doc/scipy/). The *p*-values were estimated using paired Student’s t-test in single dimensional statistical analysis.

Metabolites that differed significantly between the CG and AT group (*p* < 0.05, VIP >1) were defined as differential metabolites (DMs). The DMs were analyzed for biochemical pathways using the Kyoto Encyclopedia of Genes and Genomes (KEGG, http://www.genome.jp/kegg/). These metabolites were classified according to the pathways they were involved or the functions they performed. Pathway enrichment analysis (performed using scipy.status [Python packages, version 1.0.0]) was carried out to determine whether a group of metabolites was in a function node.

## 3 Results

### 3.1 Histological and biochemical response of *Laminaria japonica* under amantadine exposure

**FIGURE 1:**
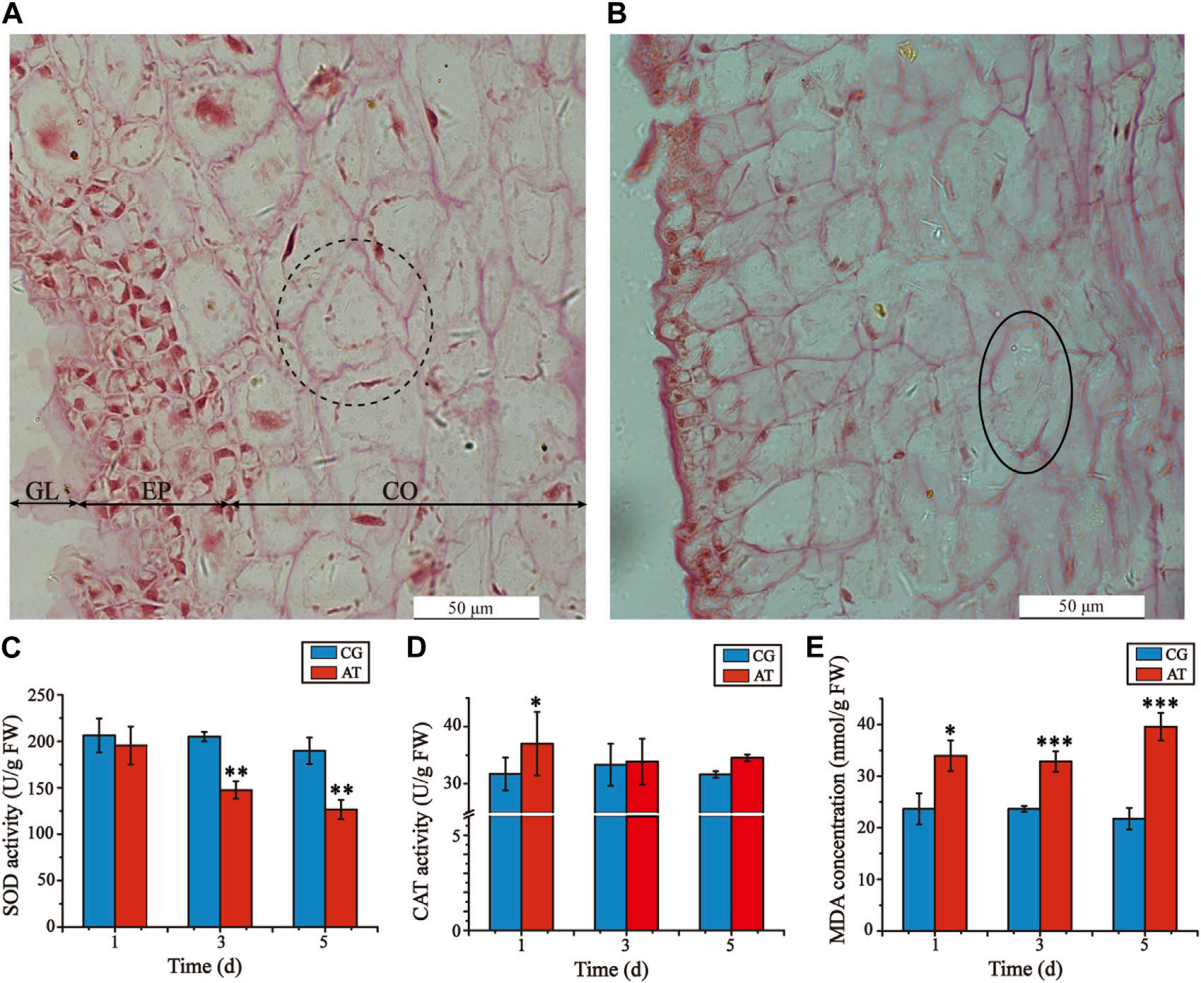
Histological alterations of juvenile sporophytes and oxidative stress response of *Laminaria japonica* under amantadine stress. **(A)** Observation of CG on the third day. **(B)** Observation of AT on the third day. GL, gelatinous layer; EP, epidermis; and CO, cortex. The black dotted circle indicates cortical cells with regular shapes. The black circles indicate cortical cell rupture. The black arrow indicates epidermis injury. Scale bar = 50 μm. **(C)** CAT activity. **(D)** SOD activity **(E)** MDA concentration. The data shown in the graph are from triplicate experiments (mean ± S.D.). Significant differences between the CG and AT group are marked as follows: *p* < 0.001 is marked as ***, *p* < 0.01 is marked as **, and *p* < 0.05 is marked as *.

Decomposition of *L. japonica* was observed after amantadine exposure for 3 days, resulting in notable variations in the cell morphology of the AT in comparison to the CG, especially the epidermal and cortical cells. The cells in the CG were uniformly stained and organized in a neat and compact manner, displaying consistent cell shapes (Figure 1A). In the AT group, the gelatinous layer was significantly thinner, the epidermal cells were absent and disorered, and the cortical cells were irregular, shrunken, and fragmented (Figure 1B). Furthermore, shallow staining and cell rupture were observed after treatment with amantadine. These results showed that amantadine had a toxic effect on *L. japonica*.

To determine the oxidative damage of *L. japonica* after amantadine exposure, the CAT activities, SOD activities, and MDA concentrations were measured on days 1, 3 and 5. The SOD activities of the AT group were lower than those of the CG group for all measurements, demonstrating a declining trend (Figure 1C). The CAT activities of the AT group were slightly higher compared to those of the CG group for the first day, after which no significant disparity was observed (Figure 1D). By comparison, the MDA concentrations of the AT group were significantly higher than those in the CG group and showed an overall increasing trend as the exposure time increased (Figure 1E). Notably, the antioxidant enzyme activities and MDA concentrations of the CG group remained stable as the exposure time increased. This suggests that amantadine was responsible for triggering lipid peroxidation.

### 3.2 Overview identification information of metabolites

**FIGURE 2:**
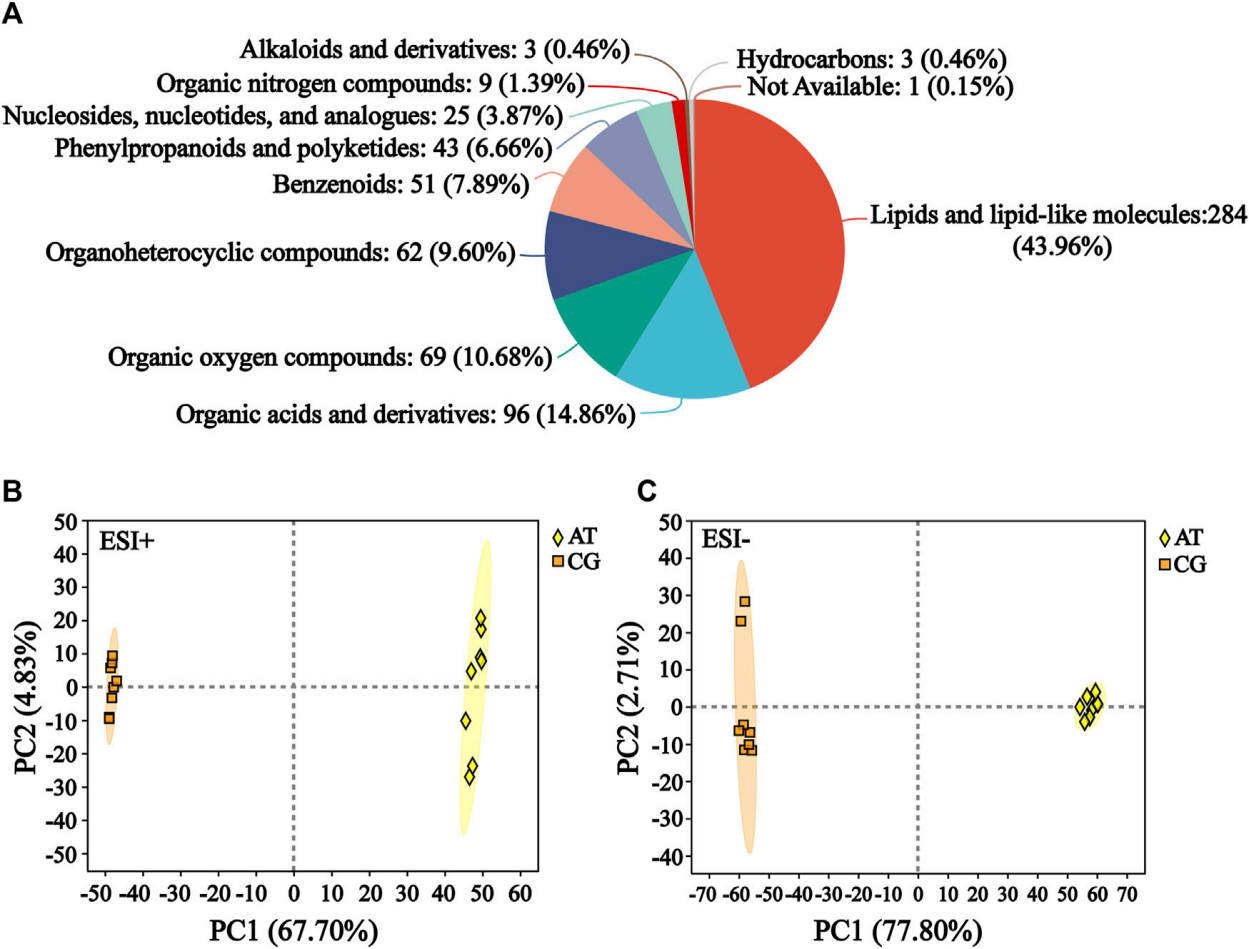
Identification of metabolites. **(A)** Compound classification of all the identified metabolites in HMDB. The different colors in each pie chart in the figure represent different HMDB categories, and the area represents the relative proportion of metabolites in that category. **(B)** Principal component analysis (PCA) of the ESI-positive ion mode (ESI+). **(C)** PCA of ESI-negative ion mode (ESI−).

The raw data of metabolomics can be found in MetaboLights (https://www.ebi.ac.uk/metabolights/) under the accession number MTBLS10548. In the total ion chromatogram diagrams of the QC samples, the peak retention times and response intensities showed extensive overlap in both the positive and negative ion modes (Supplementary Figure S1), which confirmed that the MS system was stable. After preprocessing, 876 compounds were identified, including 627 in positive ion mode and 249 in negative ion mode. The compound classification of the identified metabolites in the HMDB was shown in Figure 2A. These metabolites were divided into more than 10 categories, with the main categories being lipids and lipid-like molecules (43.96%), organic acids and derivatives (14.86%), and organic oxygen compounds (10.68%).

Subsequently, the differences between the CG and AT groups were assessed by PCA. In positive ion mode, the contributions of principal components 1 and 2 were 67.70% and 4.83%, respectively (Figure 2B). In negative ion mode, the contributions of principal components 1 and 2 were 77.80% and 2.71%, respectively (Figure 2C). The sum of the contributions of the two principal components exceeded 70% in both positive and negative modes. This indicated that the two principal components reflected the main characteristic information of samples. PLS-DA and OPLS-DA were performed to investigate the differences between the CG and AT group (Supplementary Figure S2). These results revealed there were significant between-group differences and intra-group aggregation, which suggested that the model did not display overfitting.

### 3.3 Analysis of differential metabolites

**FIGURE 3:**
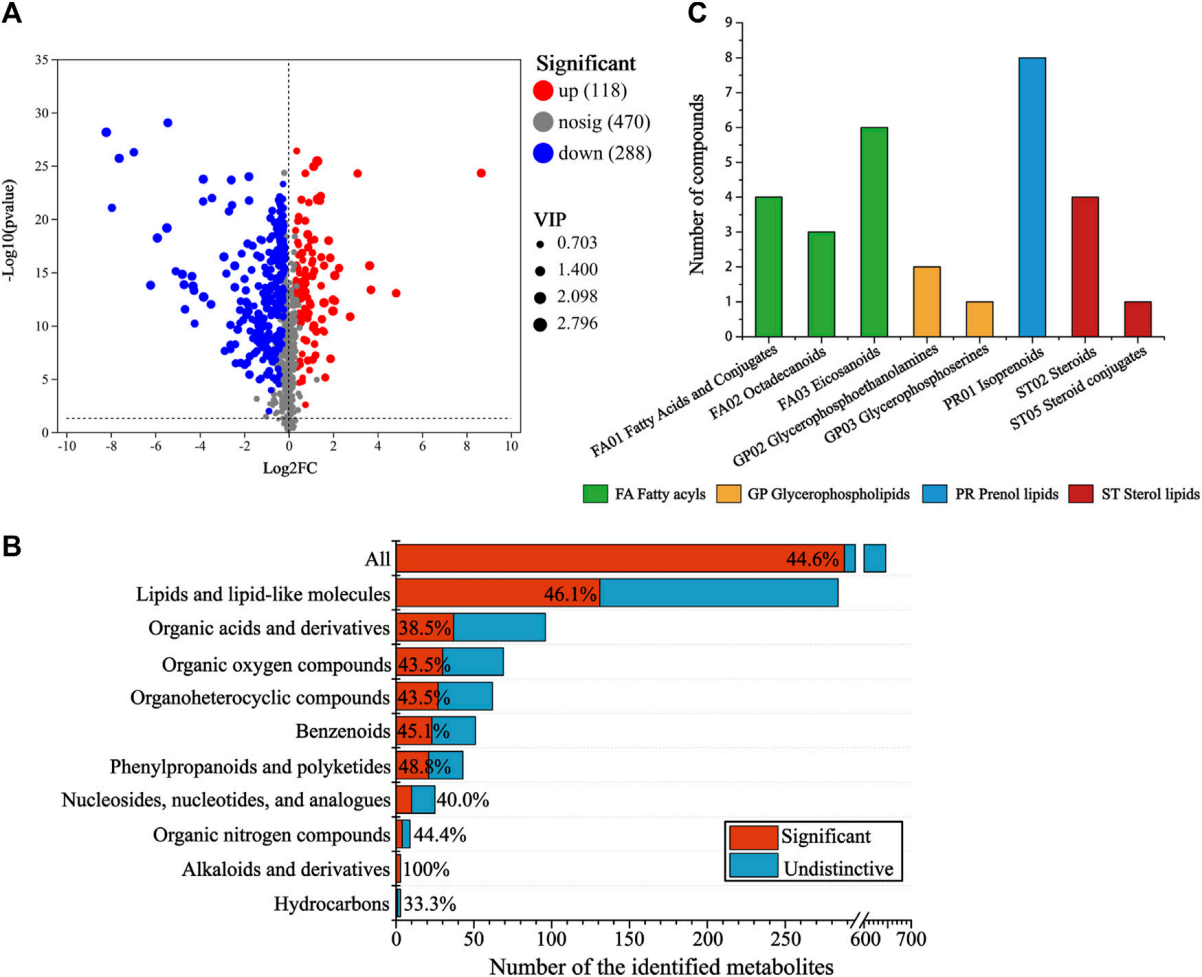
Analysis of differential metabolites (DMs) between the control and amantadine treatment group. **(A)** Volcano map of up- and downregulated DMs. **(B)** Proportion of DMs *versus* identified metabolites after amantadine exposure among different super classes in the HMDB. For each superclass, the length of the bar indicates the total number of metabolites and the red region indicates the significantly (*p* < 0.05) changed metabolites (DMs). **(C)** KEGG compounds classification of DMs. The colors of the columns represent the first category, and the *x*-axis shows the second category of KEGG compounds.

To screen the differential metabolites (DMs) between the CG and AT group, single-factor analysis and multivariate statistical analysis were conducted using Student’s t-test (*p* < 0.05) and VIP scores >1. In total, 406 significantly changed metabolites were annotated (Figure 3A), 118 metabolites were differentially upregulated and 288 metabolites were downregulated. To determine the distribution of changes among various metabolite super classes in the HMDB database, the proportion of DMs was plotted against the identified metabolites (Figure 3B). In total, 44.6% of the identified metabolites were differentially expressed after amantadine treatment. Many metabolites of lipids and lipid-like molecules significantly changed (46.1%). Other super classes with many DMs were the organic acids and derivatives (37 DMs), and organic oxygen compounds (30 DMs). The statistically significant DMs identified in the KEGG compounds classification were shown in Figure 3C. There were 29 metabolites in the lipids group, including 13 fatty acyls (green column), 3 glycerophospholipids (orange column), eight prenol lipids (blue column), and five sterol lipids (red column).

**FIGURE 4:**
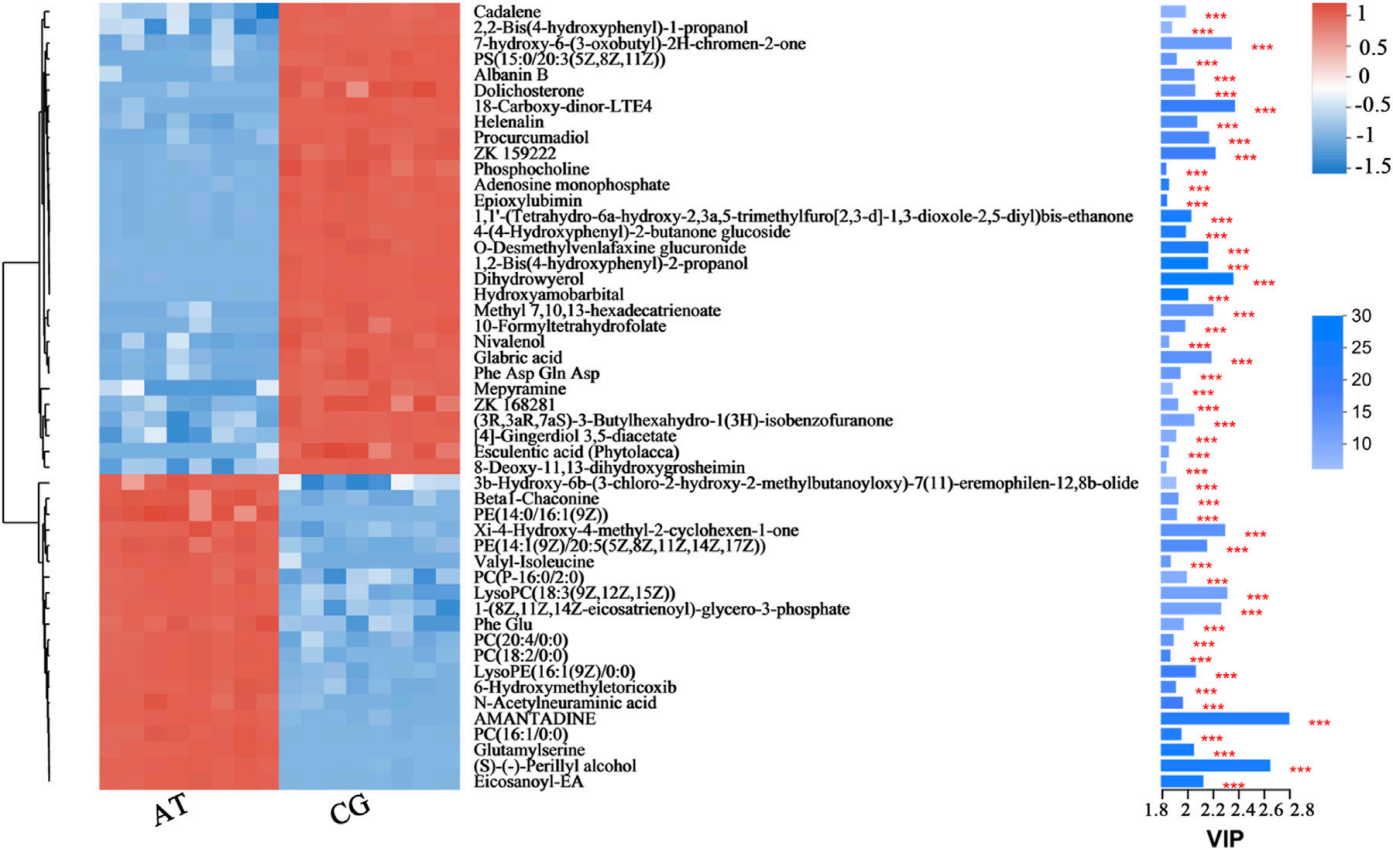
Fifty metabolites with the highest VIP spectrograms in hierarchical cluster analysis. The metabolite cluster dendrogram is shown on the left. The closer the branches, the closer the expression patterns of all metabolites in different groups. The VIP bar graph of the metabolites is shown on the right. The length of the bar represents the contribution of the metabolite to the difference between the two groups. The default value is not less than one. The colors in the figure represent the relative expression level of the metabolites (red: higher expression; and blue: lower expression). *** represents the significant difference at *p* < 0.001.

From hierarchical cluster analysis and the VIP scores, coupled with Student’s t-test (*p* < 0.05) results, the degree of correlation between metabolites and trends in expression changes were detected. Notable differences were observed between metabolites from the CG and AT groups (Figure 4). Most of the metabolites identified with significantly different levels were annotated as lipids and amino acids. For instance, the differential expression of leukotriene E4 (LTE4), prostaglandin H2 and 12-oxo-eicosatetraenoic acid (12-oxo-ETE) were observed, which are related to arachidonic acid metabolism. The expression of phospholipids was also significantly changed, including for phosphatidylcholine (PC), phosphatidylethanolamine (PE), phosphatidylserine (PS), lysophosphatidylcholine (lysoPC), and lysophosphatidylethanolamine (lysoPE). Additionally, amino acids and peptides (e.g., valyl-isoleucine, Phe Glu, and glutamylserine) were significantly upregulated in the AT group.

### 3.4 Metabolic pathway regulated by amantadine in *Laminaria japonica*

**FIGURE 5:**
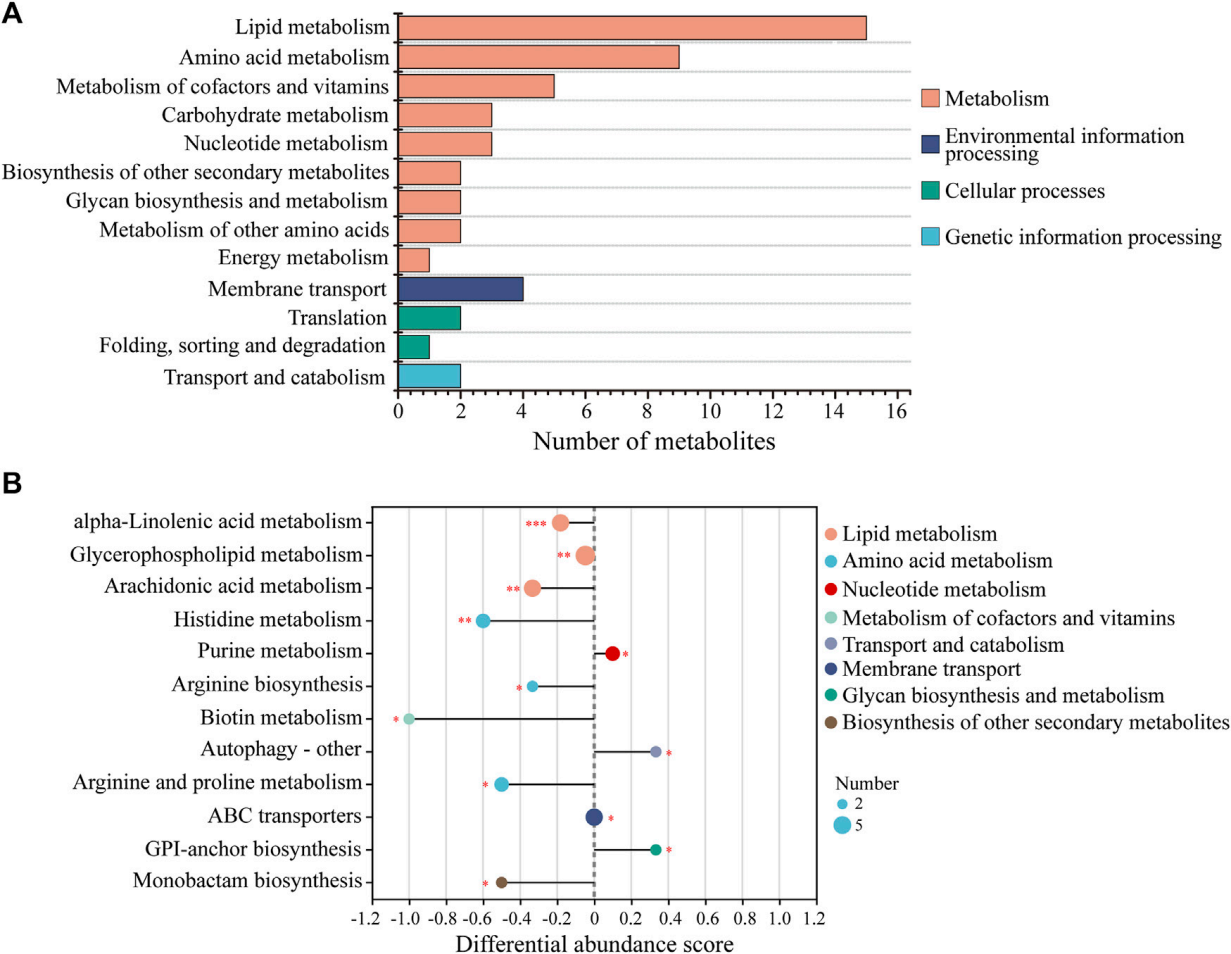
KEGG functional analysis of DMs in *Laminaria japonica* exposed to the amantadine treatment. **(A)** KEGG classification analysis. The *y*-axis shows the second category of the KEGG metabolic pathway. The *x*-axis shows the number of metabolites affected by amantadine exposure. **(B)** Differential abundance (DA) score of the enriched KEGG metabolic pathway. A DA score of 1 or -1 indicates that all the DMs annotated in this pathway were either upregulated or downregulated, respectively. *P* < 0.001 is marked as ***, *p* < 0.01 is marked as **, and *p* < 0.05 is marked as *.

**FIGURE 6:**
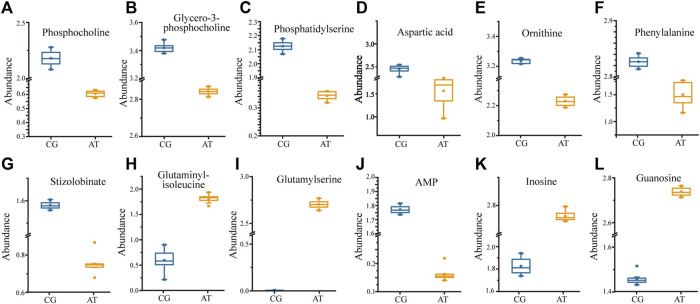
Significant regulation of metabolites (*p* < 0.05) in *Laminaria japonica* exposed to 10^7^ ng/L amantadine. The metabolites above participate in specific pathways. **(A–C)** Glycerophospholipid metabolism. **(D–I)** Amino acid metabolism. **(J–L)** Purine metabolism.

KEGG analysis of the DMs revealed the functional pathways of *L. japonica* in response to amantadine stress. Notably, most of the DMs were involved in lipid metabolism, amino acid metabolism, and metabolism of cofactors and vitamins (Figure 5A). Different metabolites may be involved in the same biological pathways, and all the annotated KEGG pathways were shown in Supplementary Table S1. Differential abundance score indicated that all the enriched lipid metabolism and amino acid metabolism were notably inhibited in response to amantadine stress, whereas nucleotide metabolism was found to be stimulated (Figure 5B). Within these enriched pathways, alpha-linolenic acid metabolism, glycerophospholipid metabolism, and arachidonic acid metabolism were significantly suppressed. Several lipid metabolites participating in glycerophospholipid metabolism were found to be downregulated, including phosphocholine, glycero-3-phosphocholine, and phosphatidylserine (Figures 6A–C). The decreased levels of amino acids were associated with pathways involving histidine metabolism, arginine biosynthesis, and arginine and proline metabolism, indicating the potential suppression of protein synthesis (Figures 5B, 6D–G). In contrast, the dipeptides containing glutamate and glutamine exhibited a notable increase under amantadine stress (Figures 6H, I). In purine metabolism, the downregulation of AMP and the upregulation of inosine and guanosine were noticed, suggesting a disruption in the synthesis of AMP (Figure 6J–L).

## 4 Discussion

**FIGURE 7:**
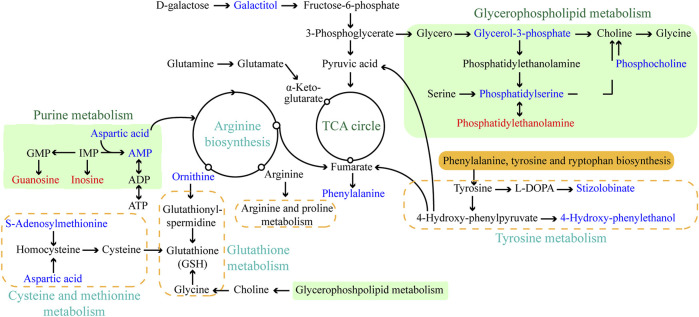
Schematic diagram of the major enriched metabolic pathways in *Laminaria japonica* exposed to 10^7^ ng/L amantadine. Arrows represent the biochemical pathways. Red and blue represent upregulated and downregulated metabolites, respectively.

The objective of this work was to comprehensively characterize the changes in histopathological and physiological characteristics and metabolic disruption for *L. japonica* caused by short-term (72 h) exposure to amantadine at a high concentration (10^7^ ng/L). The metabolites produced by *L. japonica* under amantadine stress were screened using untargeted metabolomics, which revealed changes at the cellular metabolism level. The metabolome data showed that amantadine significantly affected metabolic pathways involved in glycerophospholipid metabolism, amino acid metabolism, and purine metabolism (*p* < 0.05, Figure 7). Similar studies on abiotic stresses in algae cells have also revealed inhibition of phospholipid metabolism, purine metabolism, amino acid metabolism, and protein synthesis (Shen et al., 2023; Yang et al., 2023).

The exposure of cells, tissues, and the extracellular matrix to harmful reactive species causes a cascade of reactions and induces activation of multiple internal defense mechanisms (enzymatic or non-enzymatic) that remove reactive species and their derivatives (Mirończuk-Chodakowska et al., 2018). Biomarkers, including both enzymatic and molecular parameters, have been widely used in environmental pollution research and ecotoxicological studies. In this study, a significant decrease of SOD activity was observed after amantadine exposure, indicating the inhibiting effect of amantadine on SOD activity. The inhibition of antioxidant enzyme SOD could lead to oxidative damage of *L. japonica* because the excessive superoxide anions could not be quickly cleared. The CAT activity increased slightly more in the AT group than in the CG group on the first day, and showed no significant difference after 5 days of amantadine exposure. The activation of CAT on the first day of exposure might be due to the conversion of superoxide anions to H_2_O_2_ and play a role in the detoxification of reactive oxygen species (ROS) in *L. japonica* under amantadine exposure (Karthik et al., 2014). On days 3 and 5, the decreased CAT activity might be caused by immune fatigue.

Oxidative reactions and cellular damage could cause a significant increase in the MDA concentration (Mazdak et al., 2020). The MDA concentrations exhibited apparent increase in the experimental group exposed to amantadine, indicated that the overproduction of ROS under amantadine stress led to oxidative damage in *L. japonica*. Similar changes in antioxidant enzyme activities and MDA concentrations have been demonstrated in several toxicity studies of environmental pollution (Wang et al., 2019; Umanzor et al., 2021; Zhao et al., 2023). The results of enzymatic activities and MDA contents revealed the oxidative stress induced by amantadine in *L. japonica*, which might result in oxidative damage to multiple cellular targets.

Lipid compounds are the main components of cellular membranes and play an important role in the maintenance of their integrity and function (Mallika et al., 2007; de Carvalho and Caramujo, 2018; Chiricozzi et al., 2021). Phospholipids are the main components of the cellular lipid bilayer and the species and composition of phospholipids play a vital role in the membrane status and function and the cell activity (Olzmann and Carvalho, 2019; Yang et al., 2023). In this study, most of the lipid compounds identified with significantly different levels were phospholipids (e.g., PC, PE, PS, lysoPC and lysoPE). Furthermore, the levels of phosphocholine, glycero-3-phosphocholine, and phosphatidylserine decreased by 72.2%, 16.9%, and 81.3%, respectively, under amantadine exposure (Figures 6A–C). These changes were consistent with the results of the inhibition of glycerophospholipid metabolism in pathway enrichment analysis (Figures 5B, 7). Therefore, all these metabolic shifts showed that amantadine induced disruption of phospholipid metabolism, which was closely correlated to the cell membrane structure. Notably, recent research has demonstrated that amantadine can interact with the cell membrane and affect the cell morphology (Suwalsky et al., 2015). This was confirmed in the histological observations, with irregular and fragmented cells observed after amantadine exposure (Figures 1A, B). Similarly, silver nanoparticles (AgNPs) induce disruption of the algal cell membrane integrity and permeability in *Chlorella vulgaris*, with associated decreases in glycerol-3-phosphate and myo-inositol (Qu et al., 2021). Consequently, amantadine exposure can disrupt or damage the cell membrane through the phospholipid metabolism in *L. japonica*. This can interfere with several intracellular processes and lead to further metabolic toxicity.

Amino acids involved in physiological processes, such as osmotic pressure regulation and energy metabolism, act as important indicators of oxidative stress (Wu et al., 2013). The changes in the levels of amino acids in this study led to alteration of amino acid-related metabolic pathways, including glutathione (GSH) metabolism, tyrosine metabolism, cysteine and methionine metabolism, and arginine biosynthesis (Figure 7). These changes demonstrated that protein synthesis was affected. The accumulation of glycine, cysteine, and ornithine promoted GSH biosynthesis and the downregulated metabolites associated with these amino acids in this study (Figures 6D, E) indicated that the GSH metabolism was affected by amantadine (Tapiero et al., 2002; Qu et al., 2021). Because GSH removes ROS and maintains the intracellular redox state, disturbance of GSH metabolism results in oxidative stress and cell damage (Diaz-Vivancos et al., 2015; Ji et al., 2020). Additionally, the level of phenylalanine decreased by 35.1% (Figure 6F), and metabolites involved in tyrosine metabolism were downregulated (Figure 6G). Phenylalanine is converted to tyrosine by phenylalanine hydroxylase, and tyrosine can repair damaged cells through enzyme promotion (Pribat et al., 2010; Tessari et al., 2010). Therefore, amantadine disturbed tyrosine metabolism, which triggered oxidative stress in *L. japonica*. The disruption of amino acid metabolism demonstrated that amantadine exposure induced inhibition of GSH metabolism and tyrosine synthesis, which led to oxidative stress and cellular damage in *L. japonica*.

In the present study, the levels of dipeptides containing glutamate and glutamine greatly increased after amantadine treatment (Figures 6H,I). Glutamate and glutamine are intermediates of the tricarboxylic acid cycle (TCA cycle) and influence the synthesis of pyruvate (Ryan et al., 2021). Because the TCA cycle is the core of the cell’s respiratory machinery (Fernie et al., 2004), these results indicated that the energy production of *L. japonica* was affected. Similar studies have shown activation of the TCA cycle by AgNPs in *C. vulgaris* (Qu et al., 2021), and increased energy production through the glycolytic and glycogenic pathways by amantadine in *A*. *japonicus* (Zhao et al., 2023). The levels of aspartic acid and AMP decreased by 53.0% and 90.8%, respectively (Figures 6D,J). Inosine and guanosine were upregulated in purine catabolism (Figures 6K–L). Purines and their derivatives have long been recognized as fundamental elements of intracellular energy homoeostasis (Huang et al., 2021). Therefore, our results suggest there is disruption of energy production. Similarly, another study found that AgNPs induced inhibition of glycerophospholipid and purine metabolism in algae (Shen et al., 2023). The energy deficiency and growth inhibition in algal cells were observed after polystyrene nanoplastics exposure (Xu et al., 2024). Our results indicate that the energy metabolism of *L. japonica* is involved in the amantadine stress response through the TCA circle and purine metabolism.

## 5 Conclusion

In this study, the adverse impacts of amantadine on juvenile sporophytes of *L. japonica* were identified by untargeted metabolomics combined with histopathological and physiological analyses. Tissue sections showed that the cell morphology was irregular and partially disintegrated after amantadine exposure, which was consistent with lipid metabolism disorders. The SOD activity significantly decreased under amantadine stress, whereas the CAT activity initially increased and then showed no difference. Notable increases in the MDA concentrations were observed, suggesting the oxidative damage caused by amantadine. Metabolomics analysis showed that amantadine mainly interfered with the lipid, amino acid, and energy metabolic pathways (Figure 7). Disturbance of phospholipid metabolism resulted in disruption of the cell membrane integrity and permeability. The variations in amino acid levels indicated that amantadine induced oxidative stress in algal cells by supressing GSH metabolism and tyrosine metabolism. Energy production was affected by amantadine through the TCA cycle and purine metabolism. This research should facilitate further multi-omics verification research, and it provides novel insight into the molecular mechanisms of *L. japonica* affected by amantadine toxicity.

## Data Availability

The datasets presented in this study can be found in online repositories. The names of the repository/repositories and accession number(s) can be found in the article/Supplementary Material.
