# Supplementary material for: Untargeted metabolomics reveals the mechanism of amantadine toxicity on Laminaria japonica
Source: Front Physiol. 2024 Jul 24;15:1448259. doi: 10.3389/fphys.2024.1448259 (PMC11303324; doi:10.3389/fphys.2024.1448259)
Supplement: Supplementary file 1 [file Table1.DOCX]

**Supplementary Table 1.** Differential metabolites (DMs) identified from the KEGG database.

| Pathway ID | Description | Numbers of DMs | Metabolites KEGG  compound ID |
| --- | --- | --- | --- |
| map00052 | Galactose metabolism | 2 | C05401;C01697 |
| map00220 | Arginine biosynthesis | 2 | C00049;C00077 |
| map00230 | Purine metabolism | 3 | C01367;C00387;C00294;C00020 |
| map00250 | Alanine, aspartate and glutamate metabolism | 1 | C00049 |
| map00260 | Glycine, serine and threonine metabolism | 2 | C00049;C02737 |
| map00261 | Monobactam biosynthesis | 2 | C00019;C00049 |
| map00270 | Cysteine and methionine metabolism | 2 | C00019;C00049 |
| map00300 | Lysine biosynthesis | 1 | C00049 |
| map00330 | Arginine and proline metabolism | 3 | C00019;C03415;C00077 |
| map00340 | Histidine metabolism | 3 | C00049;C20522;C02741 |
| map00350 | Tyrosine metabolism | 2 | C06044;C06048 |
| map00410 | beta-Alanine metabolism | 1 | C00049 |
| map00460 | Cyanoamino acid metabolism | 1 | C00049 |
| map00480 | Glutathione metabolism | 1 | C00077 |
| map00520 | Amino sugar and nucleotide sugar metabolism | 1 | C00270 |
| map00561 | Glycerolipid metabolism | 1 | C05401 |
| map00563 | Glycosylphosphatidylinositol (GPI)-anchor biosynthesis | 2 | C00350 |
| map00564 | Glycerophospholipid metabolism | 5 | C00670;C00588;C02737;C00350 |
| map00565 | Ether lipid metabolism | 1 | C00670 |
| map00590 | Arachidonic acid metabolism | 4 | C06462;C14807;C00427;C14717 |
| map00592 | alpha-Linolenic acid metabolism | 4 | C16308;C16321;C11512;C16300 |
| map00670 | One carbon pool by folate | 1 | C00234 |
| map00710 | Carbon fixation in photosynthetic organisms | 1 | C00049 |
| map00760 | Nicotinate and nicotinamide metabolism | 2 | C00153;C00049 |
| map00770 | Pantothenate and CoA biosynthesis | 1 | C00049 |
| map00780 | Biotin metabolism | 2 | C05552;C05921 |
| map00970 | Aminoacyl-tRNA biosynthesis | 2 | C00049;C00234 |
| map01040 | Biosynthesis of unsaturated fatty acids | 1 | C01595 |
| map01200 | Carbon metabolism | 2 | C00049;C00234 |
| map01210 | 2-Oxocarboxylic acid metabolism | 2 | C00049;C00077 |
| map01230 | Biosynthesis of amino acids | 4 | C00019;C00049;C00077;C02741 |
| map01240 | Biosynthesis of cofactors | 5 | C00019;C00153;C00234;C00020;C00049 |
| map02010 | ABC transporters | 4 | C00049;C00387;C00294;C00077 |
| map04122 | Sulfur relay system | 1 | C00019 |
| map04136 | Autophagy - other | 2 | C00350 |
| map05140 | Leishmaniasis | 1 | C02737 |
| map05146 | Amoebiasis | 1 | C02737 |
| map01100 | Metabolic pathways | 29 | C00588;C00387;C00020;C00294;C06082;C02737;C05921;C05141;C04717;C00270;C06044;C01367;C07083;C00234;C06758;C01697;C00019;C00153;C00077;C00350;C05552;C00427;C06462;C13629;C01595;C02741;C07208;C00049;C03415 |
| map01110 | Biosynthesis of secondary metabolites | 14 | C16321;C02737;C00020;C00019;C02741;C02452;C00077;C00350;C06082;C11512;C16300;C00049;C16308 |
