# Supplementary material for: Untargeted metabolomics reveals the mechanism of amantadine toxicity on Laminaria japonica
Source: Front Physiol. 2024 Jul 24;15:1448259. doi: 10.3389/fphys.2024.1448259 (PMC11303324; doi:10.3389/fphys.2024.1448259)
Supplement: Supplementary file 2 [file Image2.PDF]

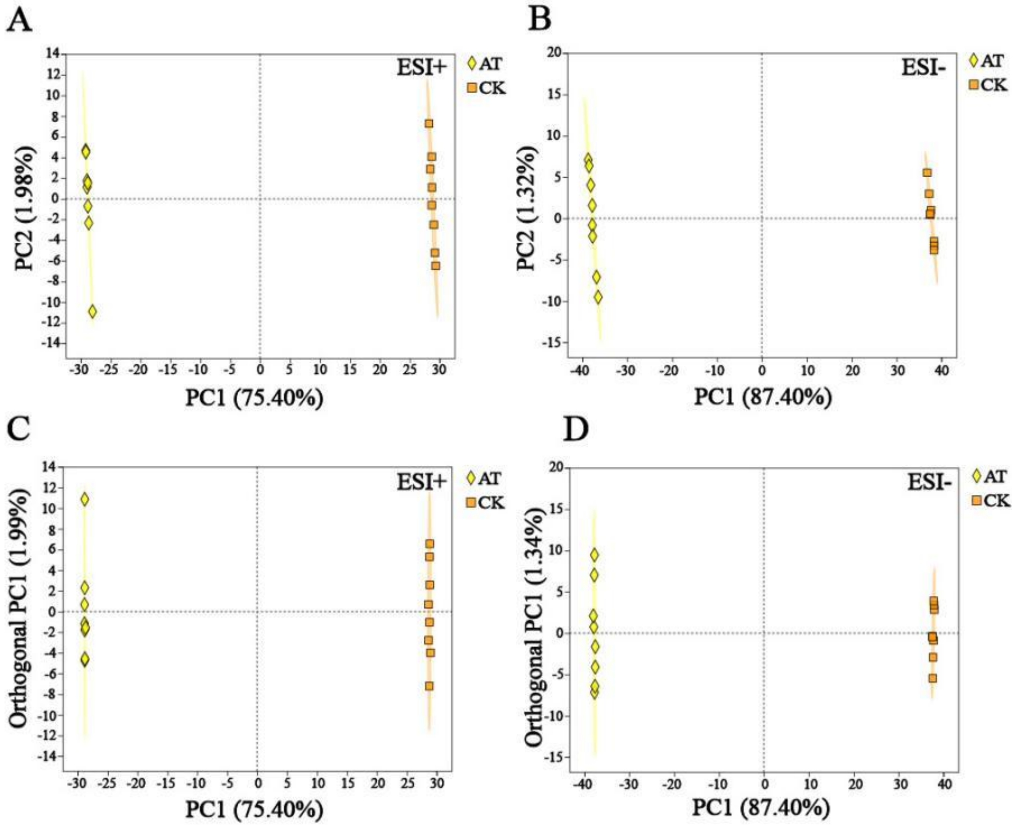

Supplementary Figure 2. Multivariate statistical analysis of amantadine in *L. japonica*. Partial least squares discriminant analysis score chart in positive (A) and negative (B) modes. Orthogonal partial least squares discriminant analysis score chart in positive (C) and negative (D) modes.
