# Supplementary material for: Untargeted metabolomics reveals the mechanism of amantadine toxicity on Laminaria japonica
Source: Front Physiol. 2024 Jul 24;15:1448259. doi: 10.3389/fphys.2024.1448259 (PMC11303324; doi:10.3389/fphys.2024.1448259)
Supplement: Supplementary file 3 [file Image1.PDF]

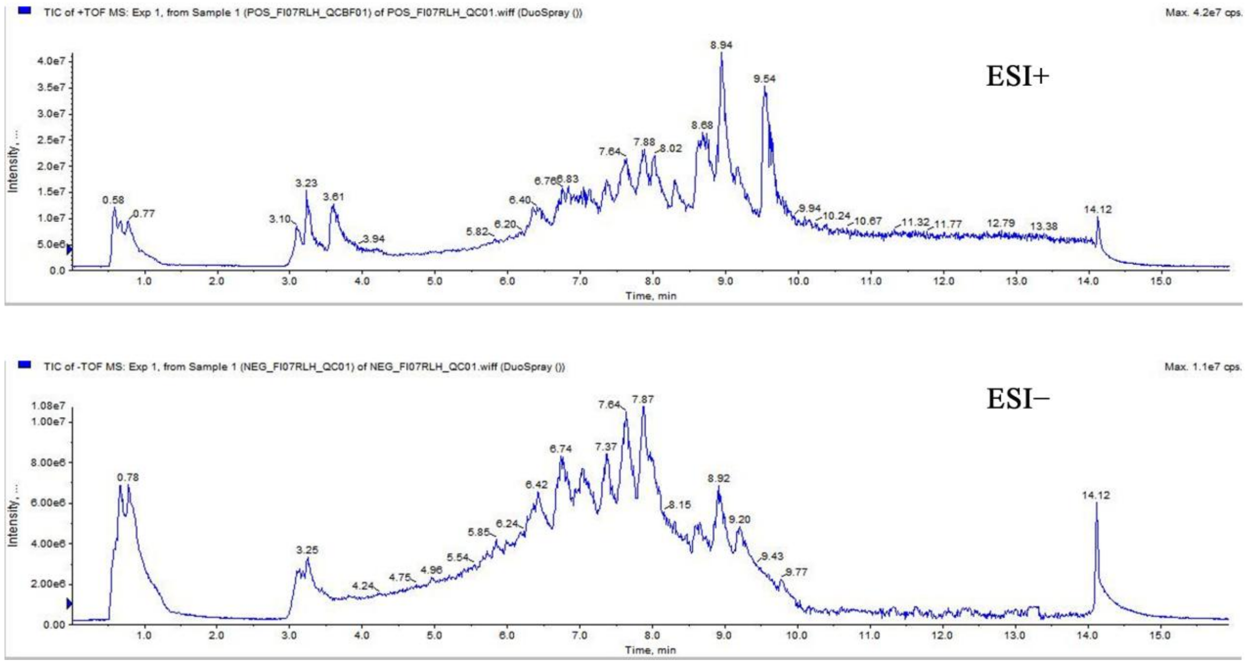

Supplementary Figure 1. Metabolomic alterations in *Laminaria japonica* following 96 h exposure to amantadine in ESI-positive ion model (ESI+) and ESI-negative ion model (ESI-).
